# Preconscious Processing Biases Predict Emotional Reactivity to Stress

**DOI:** 10.1016/j.biopsych.2009.11.018

**Published:** 2010-02-15

**Authors:** Elaine Fox, Shanna Cahill, Konstantina Zougkou

**Affiliations:** Department of Psychology, University of Essex, Colchester, United Kingdom

**Keywords:** Anxiety, attention, cortisol response, emotion, processing bias, stress

## Abstract

**Background:**

Anxiety vulnerability is associated with biases in attention: a tendency to selectively process negative relative to neutral or positive information. It is not clear whether this bias is: 1) related to the physiological response to stressful events, and 2) causally related to the development of anxiety disorders.

**Methods:**

We tested the predictive value of both preconscious and conscious attention biases in a prospective study of stress reactivity in a nonclinical sample. One hundred four male participants were assessed at baseline and then again 4 months (*n* = 82) and 8 months later (*n* = 70). Salivary cortisol and self-report measures were obtained at the baseline testing session in addition to measures of biased attention. Subsequent emotional reactivity was assessed by means of salivary cortisol and self-reported state-anxiety responses during a laboratory-based stressor (4 months later) as well as during a real-life stressor 8 months later (i.e., examination period).

**Results:**

Regression analyses indicated that a preconscious negative processing bias was the best predictor of the cortisol response to stressful events. Importantly, a measure of selective processing provided a better indicator of subsequent emotional reactivity than self-report measures of neuroticism, trait-anxiety, and extraversion.

**Conclusions:**

These results suggest that preconscious biases toward negative material play a causal role in heightened anxiety vulnerability. Our results illustrate the potential utility of preconscious biases in attention in providing an early marker of anxiety vulnerability and a potential target for treatment intervention.

Exaggerated emotional responses to stressful situations are a marker of increased vulnerability to anxiety, but remarkably little is known about the underlying mechanisms (1). Clinical diagnosis is based largely on subjective criteria, and self-report measures of personality traits such as neuroticism are the most common risk factors examined in anxiety research. Neuroticism is a higher-order personality dimension known to have a significant albeit complicated genetic component (2,3). With broad effects on mood, cognition, and neurobiological processes (4), longitudinal studies have shown that neuroticism is an established risk factor for a range of affective disorders (5,6).

Other research has focused on selective processing biases as risk factors for affective disorders. In particular, attentional processes operating early in the stream of information processing are thought to play an important role in the maintenance and causation of anxiety-related problems. Enhanced vigilance for threat at an early stage speeds up the initial perception of threat, leaving a person more vulnerable to anxiety and stress-related problems. This is probably because an early bias to process threat activates the hypothalamic-pituitary-adrenocortical (HPA) axis, leading to an increase in circulating glucocorticoids, such as cortisol. Indeed, a tendency to selectively process threatening facial expressions rather than smiling faces has been associated with enhanced cortisol release in humans (7). Biases to be vigilant for negative rather than positive information are typically measured by differences in the speed of responding to probe stimuli occurring in a location previously occupied by negative stimuli, relative to locations previously occupied by neutral or positive stimuli. Such “visual probe” tasks (VPTs) show evidence for selective processing of threat across all the major anxiety disorders—generalized anxiety disorder (GAD), obsessive-compulsive disorder, panic disorder, social phobia, and posttraumatic stress disorder—as well as in nonclinical groups who report high levels of neuroticism or trait-anxiety (8–10). Thus, much of the research indicating that neuroticism predisposes to anxiety might actually reflect the role of selective biases in attention as the key risk factor.

There is some support for this hypothesis in that persistent biases for threat are significantly reduced and sometimes eliminated by successful cognitive behavioral therapy in GAD (11) and social phobia (12), and the reduction in worry—a key feature of GAD –correlates with the magnitude of the reduction in bias (13). Laboratory research has also shown that reducing the magnitude of selective processing biases can influence emotional reactivity to subsequent stress in nonclinical groups (7,14) and predicts a reduction in anxiety symptoms in social phobia (15) and GAD (16) patients. These results are the first evidence that selective processing biases might play a causal role in the development of anxiety disorders and are therefore important targets for therapeutic intervention (17). There are surprisingly few prospective studies investigating the impact of selective processing biases on reactivity to subsequent stressful life events. Moreover, to our knowledge no prospective studies have examined the impact of selective processing biases on the physiological response to subsequent stress, although experimentally reducing bias has been shown to reduce the physiological response to work-related stress (7).

The hypothesis addressed in this study is that the magnitude of attentional bias might be a useful cognitive marker of emotional reactivity, which is associated with the development and maintenance of abnormal anxiety states. Selective processing biases based on reaction time differences to locations occupied by positive and negative stimuli provide a far more specific measure than more general self-report (e.g., neuroticism) or physiological measures (e.g., amygdala reactivity to threat). Although studies using functional magnetic resonance imaging (fMRI) have shown that the amygdala is highly reactive to threat-related stimuli (18), for instance, this is not an ideal indicator of anxiety vulnerability, because the amygdala and associated circuits also react to a range of novel and affectively positive stimuli (19,20). Thus, biased attention might be a better endophenotype, because processing biases are known to be uniquely associated with increased anxiety vulnerability (8,9,14).

Selective processing of threat cues is not simply a behavioral marker of anxiety disorders; rather it provides a window into the cognitive mechanisms associated with anxiety vulnerability (8,21–23) that in turn is a risk factor for the development of anxiety disorders. Thus, measures of biased attention might provide an important step forward in the search for early predictors of anxiety vulnerability and stress-related problems. A small number of prospective studies have shown that selective processing of threat predicts subsequent stress reactivity. For instance, a recent study (24) investigated the predictive value of skin conductance response (SCR) to two categories of pictures: negative-high arousal and pleasant-low arousal in a sample of police recruits, and reactivity to stress was assessed by means of a subjective scale administered 24 months later. The SCR reactivity to masked (but not unmasked) negative pictures was a strong predictor of subsequent distress. Masking involves the rapid replacement (i.e., 14–30 msec) of experimental stimuli with a meaningless one to prevent conscious awareness of the stimulus. Several studies show that biased attention for threat provides a good indicator of subsequent distress but only when the critical stimuli are masked. In clinical populations, for example, threat biases under masked conditions have consistently been found, suggesting that these biases emerge automatically and play a crucial role in the etiology of anxiety disorders (21–23). A small number of prospective studies support a causal role for preconscious processing biases in showing that they predict later distress in patients awaiting colposcopy after a positive cervical smear test (25), in women undergoing treatment for infertility (26), in academically stressful situations (27), in laboratory-based stress tasks (28), and in laboratory-induced stress caused by inhalation of carbon dioxide–enriched air (29).

These studies provide important information on the role of selective processing biases in the cognitive mechanisms underlying anxiety vulnerability. However, all of these studies have focused exclusively on subjective measures of stress reactivity. It is well-established that activation of the HPA axis—as indexed by levels of circulating cortisol—is a good physiological marker of stress. Increased cortisol release is elicited reliably by acute stressful situations (30), is characteristic of many clinical anxiety states (31), and is associated with a negative processing bias in attention (7). A novelty of the current study is that a physiological indicator of stress (cortisol response) was used in a prospective study in addition to subjective measures to investigate emotional reactivity in nonclinical participants in both laboratory-based and more realistic (examination stress) stressful situations.

## Methods and Materials

### Participants

One hundred four male participants were recruited from the University of Essex during the autumn term (October–November) of 2004. All were between 18 and 30 years of age and gave informed consent to take part in the study. Eighty-two of these participants were retested approximately 4 months later (January–February 2005), and 70 of the original 104 were tested for a final time approximately 8 months after the initial baseline session (May–June 2005). The study was approved by the University of Essex Ethics Committee.

### Materials

#### VPT

Selective attention to negative and positive images was determined by means of a pictorial VPT task. Twenty negative, 20 positive, and 40 neutral pictures were selected from the International Affective Picture System (IAPS) (32), with the affective pictures being matched for arousal. Each trial of the experiment presented two pictures above and below a central fixation point. One of the pictures was affectively salient (either positive or negative), and the other was always neutral. All pictures were presented in gray scale and measured 3.5 × 4 cm with a distance of 4 cm from the central fixation to the center of each picture. At a standard viewing distance of 60 cm this gave a distance of 3.8 degrees of visual angle from fixation to the center of each picture. Target stimuli consisted of two dots at either vertical (:) or horizontal (..) orientation measuring .5 cm and were presented in the center of either the upper or lower location. Valence of picture and location of target were counterbalanced across the experiment. A masking stimulus was also constructed from randomly cut and reassembled portions of a selection of the gray scale pictures used in the experiment. This stimulus completely covered the IAPS pictures.

All participants were tested in a quiet, dimly lit room, and a chin rest was placed 60 cm from the center of a computer screen presented at eye-level. All stimuli were presented on a high-quality 17-inch monitor with a resolution of 768 × 1024 pixels and connected to a Power Macintosh computer running PsyScope experimental software (33). After a short set of practice trials a set of 320 experimental trials were presented with a break after every 80 trials. Each trial consisted of a fixation presented at the center of the screen for 500 msec, followed by a display of two pictures and a central fixation for either 300 msec (aware) or 14 msec (unaware); a masking display consisting of a central fixation and the masking stimulus presented both above and below fixation in the location of the previous pictures was then presented for either 200 msec or for 486 msec. Finally, the target stimulus (: or ..) was presented at the location of either the upper or lower picture until response. One-half of the trials (*n* = 160) were masked after 14 msec, whereas one-half were masked after 300 msec (160). Each set of aware and unaware trials consisted of equal numbers of positive-neutral and negative-neutral picture combinations (80). Each participant was presented with a different randomized order of trials. The participant's task was to respond to the target stimulus by pressing either the left- or the right-hand button on a specially designed key-pad. The left or right key was assigned to the “:” target for one-half of the participants and to the “..” target for the other half.

The VPT provided four measures of attentional bias: aware and unaware negative bias scores (i.e., the mean individual reaction times to probes occurring in the location of neutral pictures minus the mean reaction times to probes occurring in the location of negative pictures) for aware and unaware trials, respectively, and aware and unaware positive bias scores (i.e., the mean individual reaction times to probes occurring in the location of neutral pictures minus the mean reaction times to probes occurring in the location of positive pictures) for aware and unaware trials, respectively. A numerically positive score for each of these measures of bias indicates vigilance for the affective stimulus, whereas a numerically negative bias score indicates selective avoidance of the affective picture.

#### Questionnaires and Rating Scales

Trait and state-anxiety were measured with the Spielberger State-Trait Anxiety Inventory (STAI) (34). The STAI is divided into two 20-item scales providing independent measures of trait-anxiety and state-anxiety. Each scale has a possible range of scores from 20 to 80, and normative means for trait-anxiety are close to 40. Trait depression was measured with the Beck Depression Inventory (35), which has a range of 0–63. The “Big Five” personality traits of neuroticism, extraversion, openness to experience, agreeableness, and conscientiousness were measured with a short-form of the NEO Personality Inventory (36). This consists of 30 statements (6 for each personality trait) rated on a 1–5 scale, giving a range of 6–30 for each trait.

#### Salivary Cortisol

Saliva samples were obtained by means of Salivette collection devices (Sarstedt, Leceister, United Kingdom) and were stored at −20°C before assaying. Salivary cortisol levels were determined by means of a competitive radio-immunoassay technique with a polyclonal anticortisol-antibody (K7348). The reference values for adults are 4–28 nmol/L.

### Procedure

#### Baseline Assessment

During the baseline assessment, informed consent was obtained, and then salivary cortisol was collected. Each participant then completed the VPT to measure their degree of bias toward both negative and positive images. After this, a variety of self-report questionnaires were completed to measure some general personality traits and other demographic characteristics.

#### The Laboratory-Based Stress Task

Four months after the baseline assessment, stress was induced under laboratory conditions by requiring each participant to prepare a 5-min speech on “Why we need statistics in psychology” that had to be presented in front of two experimenters and a video camera. Measures of cortisol were taken on 6 occasions: at 20 and 10 min before the speech (−20 and −10); and then at 1, 5, 10, and 20 min after the speech (+1, +5, +10 and +20). State-anxiety was measured with the state form of the STAI at −20 and +5. All participants were tested during the morning (from 9:00 am to 11:30 am) to control for diurnal variation in cortisol response.

#### The Realistic Stress Task

The realistic stress task took place 4 months later (8 months after the baseline assessment), approximately 3–6 days before important end of year examinations. All participants were tested during the morning (9:00–11:30 am) and were required to present a short talk to one experimenter and a video camera on “Have I prepared well enough for my exams?” Cortisol was measured 10 min before the speech (−10) and then at +1, +5, and +10 after the speech. State anxiety was measured at −10 and +5.

## Results

### Baseline Results

The mean age of the men was 21.8 years, and all self-report measures as well as baseline cortisol were within the normal range (Table 1).Table 2 gives the mean reaction times on the masked and unmasked VPT. The *t* tests on mean differences between valid and invalid trials did not show any overall significant differences (i.e., biases) for either positive or negative images whether they were masked or unmasked. However, as expected there was a wide range of bias scores, ranging from strong avoidance of affective pictures (−103 msec) to a strong vigilance for affective pictures (+130 msec).

Pearson correlation coefficients were calculated to determine whether masked and unmasked attentional biases correlated with subjective measures of neuroticism, extraversion, depression, and trait- and state-anxiety at baseline (Table 3). The only significant correlations were between the masked negative bias score and neuroticism [*r*(102) = .22, *p* < .05] and the masked positive bias score and neuroticism [*r*(102) = −22, *p* < .05]. This pattern indicates that higher levels of neuroticism were associated with preconscious biases to selectively process negative material and to avoid positive material. The masked negative bias was also positively correlated with the baseline level of salivary cortisol [*r*(102) = .27, *p* < .01].

### Response to Laboratory-Based Stressor

**Figure 1:**
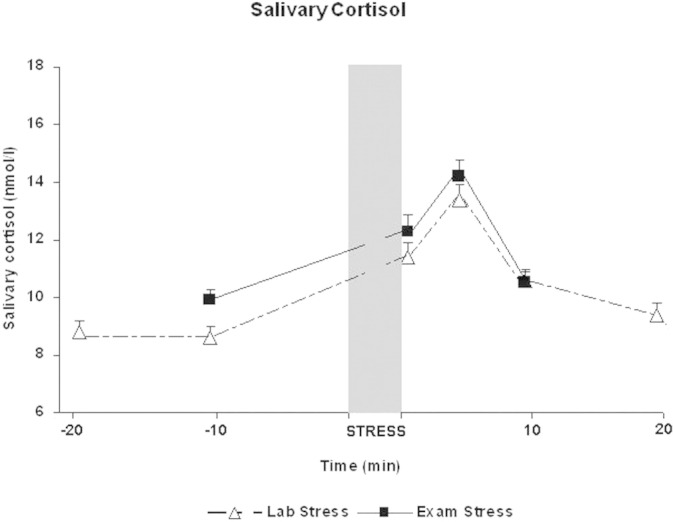
Mean level of salivary cortisol (nmol/L) at each assessment period from 20 min before the stressor (−20) to 20 min after the stressor (+20) for both laboratory- and examination-based stressful situations.

**Figure 2:**
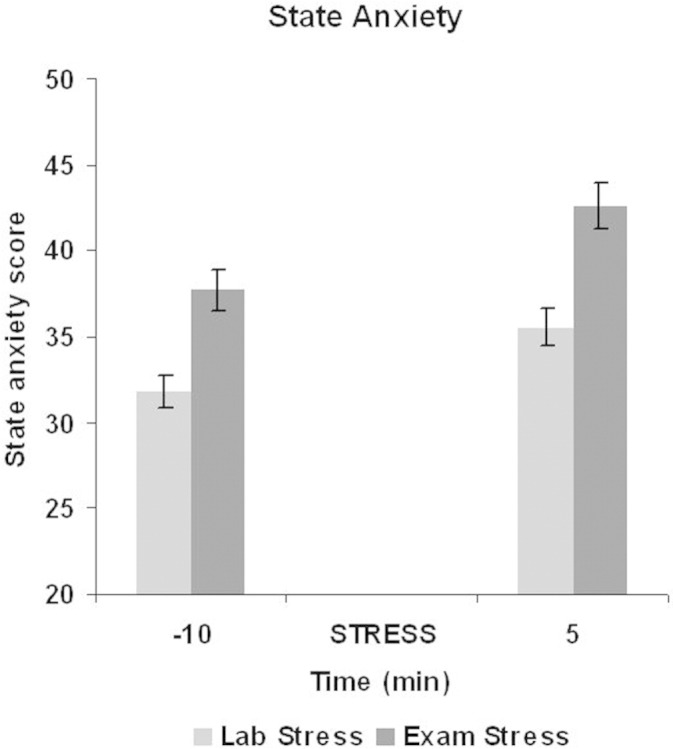
Mean level of self-reported state-anxiety at 10 min before a stressor (−10) and 5 min after a stressor (+5) for both laboratory- and examination-based stressful situations.

Figure 1 shows the mean level of salivary cortisol (nmol/L) at each of the six assessment periods. An analysis of variance revealed a significant change across these assessment periods, [*F*(5,405) = 104.7, mean square error (MSE) = 2.55, *p* < .001, ηp(2) = .56]. Planned *t* tests showed the expected increase in salivary cortisol from −20 to both +1 [*t*(81) = 9.6, *p* < .001, d = 1.06] and +5 [*t*(81) = 11.8, *p* < .001, d = 1.31] and from −10 to both +1 [*t*(81) = 10.9, *p* < .001, d = 1.21] and +5 [*t*(81) = 12.6, *p* < .001, d = 1.39]. Salivary cortisol peaked at 5 min after the stress task (mean = 13.4, SD = 5.0) and decreased significantly from this peak to the assessment made at +10 [*t*(81) = 12.0, *p* < .001, d = 1.32] and +20 [*t*(81) = 13.7, *p* < .001, d = 1.52]. All of these differences are significant after Bonferroni corrections. State-anxiety also increased significantly from −20 to +5 [*t*(82) = 4.2, *p* < .001, d = .46] (Figure 2). Thus, the laboratory-based stressor successfully elevated the cortisol response and state-anxiety.

### Response to Realistic Examination-Based Stressor

As expected, the level of subjective anxiety increased from the baseline assessment (mean = 31.3, SD = 8.8) to the initial assessment (−10) during the examination-based stressor [mean = 37.8, SD = 10.4: *t*(69) = 6.6, *p* < .001, d = .79] as did salivary cortisol [baseline mean = 8.3 nmol/L, SD = 3.9; −10 mean = 9.9 nmol/L, SD = 3.5: *t*(69) = 5.8, *p* < .001, d = .69], confirming that the examination period induced a significant degree of real-life stress in this student population. As shown in Figures 1 and 2, the examination-based stressor further increased the level of both subjective anxiety and cortisol response. For the cortisol response, an analysis of variance showed a significant difference across the four assessment periods [*F*(3,207) = 89.5, MSE = 2.9, *p* < .001, ηp(2) = .57]. As expected, salivary cortisol increased significantly from −10 to both +1 [*t*(69) = 8.5, *p* < .001, d = 1.01] and +5 [*t*(69) = 10.7, *p* < .001, d = 1.28] with a peak at +5 (mean = 14.2, SD = 5.2) before decreasing significantly at the final assessment period [+10: *t*(69) = 13.2, *p* < .001, d = 1.36]. State-anxiety also increased significantly from −10 (mean = 37.7, SD = 10.4) to +5 [mean = 42.6, SD = 11.1: *t*(69) = 4.6, *p* < .001, d = .55] (Figure 2). Thus, the examination-based stressor was successful in increasing an already elevated level of salivary cortisol and state-anxiety. All differences were significant after Bonferroni corrections.

### Prospective Relationships Between Baseline Measures and Physiological and Subjective Response to Stress

Table 3 shows the correlations among the baseline measures and the measures of salivary cortisol and state-anxiety at the various assessment points during the laboratory and examination-based stress periods. To identify the predictive value of baseline subjective reports, cortisol level, and attentional bias, a series of regression analyses were conducted. First, for both the laboratory-based stressor and the examination-based stressor, a cortisol response index was calculated by subtracting the mean salivary cortisol at the peak response time (at +5) from the mean salivary cortisol level at the −10 assessment period. This response index was used as the dependent variable, and a hierarchical regression analysis was conducted. For the laboratory stress, salivary cortisol, neuroticism, extraversion, trait-anxiety, state-anxiety, and Beck Depression Inventory scores at baseline were all entered in Step 1. The unmasked bias scores for both negative and positive images were entered in Step 2, and the masked bias scores were entered in Step 3. The results of the regression analyses for the cortisol response and for the state-anxiety response are shown in Tables 4 and 5, respectively. Model 3 explained a significant amount of the variance in the magnitude of the cortisol response to a laboratory stressor, but the only significant predictor was the masked negative attentional bias. This indicates that a preconscious bias to selectively process negative images predicted the magnitude of the salivary cortisol response to stress 4 months later. Similar results were found when cortisol reactivity was indexed by the “area under the curve” (AUC), which measures increases above an individual's baseline in response to a stressor (37). Correlations between AUC and bias scores showed that the only significant association was with the masked negative bias [*r*(80) = .425, *p* < .001]. In the regression analysis for the subjective response to laboratory stress, the strongest predictors were high neuroticism and extraversion scores along with a preconscious tendency to avoid positive images.

Tables 6 and 7 show similar analyses for the examination-based stressor. For the cortisol response to stress, the results replicated those found with the laboratory-based stressor. Model 3 explained a significant amount of the variance, with the masked negative bias being the only baseline measure that predicted the cortisol response to examination stress 8 months later. Correlations between AUC—indexed to baseline—and bias measures showed that the only significant correlation was with masked negative bias [*r*(68) = .475, *p* < .001]. In contrast, Model 3 did not significantly predict the variance in the subjective state-anxiety response to examination-based stress.

## Discussion

We present the first evidence that the preconscious tendency to selectively process negative material is associated with the magnitude of the physiological response to stress up to 8 months later in a population of healthy male undergraduate students. Preconscious bias for negative images was the only baseline indicator that significantly predicted the magnitude of the cortisol response to subsequent stress. In sharp contrast, self-report measures of neuroticism, trait-anxiety, or depression did not predict subsequent cortisol reactivity to stress. For the laboratory-based stressor, however, higher baseline levels of self-reported neuroticism and extraversion did predict a greater change in the subjective response to stress. Reduced attention for positive material at the preconscious level at baseline was also associated with a larger subjective response to a laboratory-based stressful situation. None of the baseline measures predicted—against expectation—the subjective response to realistic stress. One possibility for this is that, because state anxiety was already elevated significantly just before end of year examinations, there might have been a ceiling effect in looking for further reactivity to stress in this student population. Additional research is required to further examine the predictors of both physiological and subjective responses to stress, perhaps by using a wider range of sensitive measures. It is also worth noting that we tested an entirely male sample to control for potential variation in cortisol release over the menstrual cycle. Therefore, replication is required in female samples as well as in clinical samples to see whether the results generalize.

Our results strongly suggest that selective processing biases in early attention are predictors of stress reactivity that might predispose to anxiety disorders. The indication is that these “on-line” measures of processing selectivity provide better indicators of anxiety vulnerability than the self-report measures of neuroticism that are widely used in psychiatric research. Future studies of treatment efficacy in anxiety disorders could therefore usefully include measures of biased attention as outcome variables. It is possible of course that our results were affected by the degree of attrition from baseline testing (*n* = 104) to the final testing session 8 months later (*n* = 70). The sample size was relatively small and a larger study might find stronger evidence for the utility of self-report measures as predictors of stress-reactivity. Nevertheless, the current results indicate that measures of processing biases in attention are important variables to include in treatment studies.

Our results support the view that preconscious biases toward negative material might increase people's vulnerability to becoming physiologically and subjectively anxious in response to stress and that such stress reactivity might in turn predispose to the development of anxiety disorders. Biased attention as measured by speed of response to negative and positive material provides more information than simply being a behavioral marker of anxiety vulnerability. These biases in attention, especially those that occur at an implicit or preconscious level provide a window into the cognitive mechanisms that underlie the development of anxiety-related problems. The evidence is now growing that preconscious selective processing biases are likely to play a causal role in the development of anxiety as well as playing a role in the maintenance of anxious mood states. Thus, simple measures of biased attention might provide an early warning of potential vulnerability to anxiety.

Selective processing of affective material has recently been shown to be associated with common variations in the serotonin transporter gene (38,39), and experimental modification of these biases results in significant changes in emotional vulnerability (17). To date, however, the scientific evidence relates to a group level of analysis rather than an individual level. Future research needs to focus on a more detailed analysis of the usefulness of individual propensities to selectively process both negative and positive material and how this relates to subsequent vulnerability and resilience to stress. Given the growing evidence that negative biases in attention are important indicators of increased anxiety vulnerability, these biases are likely to be reliable early warning signs and might also be appropriate targets for therapeutic interventions. Online measures of processing biases are likely to form an important element in the chain from individual genes to complex human conditions, such as anxiety disorders.

## Figures and Tables

**Table 1:** Means, SD, and Range for All Measures Taken for 104 Participants at the Baseline Testing Session

	Mean	SD	Range
Age, yrs	21.8	2.9	18–30
Trait Anxiety	40.1	9.6	22–74
Beck Depression Inventory	7.7	6.9	0–40
State Anxiety	32.0	8.2	20–67
Neuroticism	16.1	5.1	6–29
Extraversion	22.8	3.5	14–29
Openness to Experience	22.6	5.0	13–30
Agreeableness	20.2	3.2	11–27
Conscientiousness	20.0	4.9	12–29
Salivary Cortisol (nmol/L)	8.2	3.8	4–22

**Table 2:** Mean RTs and SEM as a Function of Masking, Validity, and Valence of Picture on the Dot-Probe Test for Attention Bias

	Masked		Unmasked	
	Valid	Invalid	Valid	Invalid
Negative Picture				
Mean RT (msec)	732.5 (12.8)	737.1 (12.4)	724.4 (11.9)	725.2 (12.6)
Bias score	4.6		.8	
Range in msec	(−103.0–100.0)		(−102.4–80.2)	
Positive Picture				
Mean RT (msec)	722.5 (12.6)	719.7 (12.8)	713.9 (12.2)	713.3 (12.3)
Bias score	−2.8		−.7	
Range in msec	(−95.7–129.8)		(−89.3–88.4)	

Means and ranges for each bias score as a function of masking and valence of face and are also presented. RT, reaction time.

**Table 3:** Correlations Between Masked and Unmasked Positive and Negative Biases, Neuroticism, Extraversion, Trait Anxiety, Depression, and State-Anxiety and Cortisol at Baseline and Immediately After Laboratory and Exam-Related Stress

	Mask NBias	Mask PBias	NoMask NBias	NoMask PBias	N	E	Trait Anx	BDI	State (T1)	Cort (T1)	State (T2)	Cort (T2)	State (T3)	Cort (T3)
Masked NBias														
Masked PBias	−.09													
Unmasked NBias	−.08	−.16												
Unmasked PBias	−.13	.15	−.21^a^											
N	.22^a^	−.22^a^	−.15	−.02										
E	.11	.22^a^	.00	.03	−.30^b^									
Trait Anxiety	.17	−.19^a^	−.01	−.02	.76^c^	−.49^c^								
BDI	.05	−.03	−.08	−.10	.64^c^	−.29^b^	.72^c^							
State-anxiety (T1)	.11	−.07	.00	.03	.51^c^	−.23^a^	.62^c^	.42^c^						
Cortisol (T1)	.27^b^	−.10	.07	−.13	.36^c^	−.17	.30^b^	.25^b^	.15					
State-anxiety (T2)	.03	−.25^a^	.12	.09	.54^c^	−.10	.47^c^	.44^c^	.66^c^	.13				
Cortisol (T2)	.46^c^	−.15	.15	−.17	.28^a^	−.11	.30^b^	.27^a^	.19	.79^c^	.15			
State-anxiety (T3)	−.04	−.12	−.23	.13	.30^a^	−.24^a^	.44^c^	.35^b^	.41^b^	−.11	.25^a^	−.03		
Cortisol (T3)	.47^c^	−.15	.03	−.09	.39^b^	−.21	.33^b^	.29^a^	.18	.76^c^	.15	.85^c^	−.06	

BDI, Beck Depression Inventory; NBias, negative bias; PBias, positive bias; T1, state-anxiety (State) and cortisol (Cort) at baseline; T2, state-anxiety and cortisol immediately after laboratory stress; T3, examination-related stress; N, neuroticism; E, extraversion.

a *p* < .05.

b *p* < .01.

c *p* < .001.

**Table 4:** Summary of Multiple Regression Analysis for the Laboratory-Based Stressor

Predictors	β	*t*	*R*^2^	Δ*R*^2^
Model 1			.029	
Model 2			.056	.027
Model 3			.212	.156^a^
State anxiety	.092	<1		
Trait anxiety	−.085	<1		
Neuroticism	−.121	<1		
Extraversion	.001	<1		
BDI	.132	<1		
Cortisol	−.051	<1		
Unmasked PBias	−.058	<1		
Unmasked NBias	−.064	<1		
Masked NBias	.453	3.7^a^		
Masked PBias	.032	<1		

The outcome measure was the change in salivary cortisol from before the stressor (−10) to the peak response that occurred after the stressor (+5). Abbreviations as in Table 3.

a *p* < .001.

**Table 5:** Summary of Multiple Regression Analysis for the Laboratory-Based Stressor

Predictors	β	*t*	*R*^2^	Δ*R*^2^
Model 1			.142	
Model 2			.174	.031
Model 3			.261	.088^a^
State anxiety	−252	<1		
Trait anxiety	−.140	<1		
Neuroticism	.392	2.3^a^		
Extraversion	.273	2.1^a^		
BDI	.082	<1		
Cortisol	−.010	<1		
Unmasked PBias	−.029	<1		
Unmasked NBias	.098	<1		
Masked NBias	−.219	1.9		
Masked PBias	−.258	−2.3^a^		

The outcome measure was the change in state-anxiety from before the stressor (−10) to 5 min after the stressor (+5). Abbreviations as in Table 3.

a *p* < .05.

**Table 6:** Summary of Multiple Regression Analysis for the Examination-Based Stressor

Predictors	β	*t*	*R*^2^	Δ*R*^2^
Model 1			.141	
Model 2			.174	.033
Model 3			.353	.179^a^
State anxiety	.027	<1		
Trait anxiety	−.085	<1		
Neuroticism	.231	<1		
Extraversion	.052	<1		
BDI	−.018	<1		
Cortisol	.071	<1		
Unmasked PBias	.068	<1		
Unmasked NBias	−.088	<1		
Masked NBias	.487	4.0^a^		
Masked PBias	.009	<1		

The outcome measure was the change in salivary cortisol from before the stressor (−10) to the peak response that occurred after the stressor (+5). Abbreviations as in Table 3.

a *p* < .001.

**Table 7:** Summary of Multiple Regression Analysis for the Examination-Based Stressor

Predictors	β	*t*	*R*^2^	Delta *R*^2^
Model 1			.122	
Model 2			.135	.013
Model 3			.175	.040
State anxiety	−217	<1		
Trait anxiety	.162	<1		
Neuroticism	−.408	2.1^a^		
Extraversion	.031	<1		
BDI	.042	<1		
Cortisol	.143	<1		
Unmasked PBias	−.028	<1		
Unmasked NBias	−.187	−1.4		
Masked NBias	−.175	−1.3		
Masked PBias	−.166	−1.3		

The outcome measure was the change in state-anxiety from before the stressor (−10) to 5 min after the stressor (+5). Abbreviations as in Table 3.

a *p* < .05.
